# Downregulation of Hsp70 inhibits apoptosis induced by sialic acid-binding lectin (leczyme)

**DOI:** 10.3892/or.2013.2814

**Published:** 2013-10-24

**Authors:** TAKEO TATSUTA, MASAHIRO HOSONO, YUKIKO OGAWA, KYOKO INAGE, SHIGEKI SUGAWARA, KAZUO NITTA

**Affiliations:** 1 Division of Cell Recognition Study, Institute of Molecular Biomembrane and Glycobiology, Tohoku Pharmaceutical University, Sendai 981-8558, Japan; 2 Divisions of Functional Morphology and Microbiology, Department of Pharmacy, Faculty of Pharmaceutical Science, Nagasaki International University, Sasebo, Nagasaki 859-3298, Japan

**Keywords:** lectin, ribonuclease, leczyme, apoptosis, Hsp70, Hsc70

## Abstract

Heat shock proteins (Hsps) are molecular chaperones that maintain homeostasis of organisms. In regards to the Hsps, many studies have investigated the structure, expression, localization and functions of Hsp70 and Hsc70 including expression in the glycosphingolipid-enriched microdomain (GEM) on the cell surface and involvement in cell death. Sialic acid-binding lectin (SBL) isolated from oocytes of *Rana catesbeiana* is a multifunctional protein which has lectin activity, ribonuclease activity and antitumor activity. SBL has potential as a new type of anticancer drug, since it causes cancer-selective induction of apoptosis by multiple signaling pathways in which RNA is its target; and the participation of the mitochondrial pathway and the endoplasmic reticulum (ER) stress-mediated pathway has been suggested. It has also been suggested that receptor(s) for SBL (SBLR) may exist in the GEM on the cell surface. In the present study, we studied the possible involvement of Hsp70 and Hsc70 in SBL-induced apoptosis. We showed that Hsp70 and Hsc70 were expressed on the P388 cell surface similar to SBLR, and their distribution in cells dramatically changed immediately prior to the execution of apoptosis following stimulation of SBL. Functional study of Hsp70 revealed that decreased expression of Hsp70 diminished the apoptosis induced by SBL. It is suggested that Hsp70 participates in the antitumor effect of SBL.

## Introduction

In response to various types of stress, cells activate a highly conserved heat shock response in which a set of heat shock proteins (Hsps) are induced. These proteins play important roles in cellular repair and protective mechanisms. There are 2 types of Hsps, i.e., stress-inducible and constitutive types. In addition, it is well known that Hsps act as molecular chaperones to maintain the homeostasis of organisms (1). Recently, much information has become available on the specific role of individual Hsps. In particular, there are many significant reports regarding Hsp70 family member proteins that are closely involved in cell death. It has been reported that Hsp70 inhibits apoptosis by hindering the activation of JNK (2), or by preventing recruitment of procaspase-9 to the Apaf-1 apoptosome (3). In contrast, Hsp70 promotes apoptosis. For example, overexpression of Hsp70 was found to enhance TCR and fas-mediated apoptotic cell death (4). Among the Hsp70 family, Hsp70 and the constitutively expressed 73-kDa heat shock cognate protein (Hsc)70 have been found to be located in the cytosol and to migrate to the nucleus after specific stress (5), but are also expressed on the cell surface and interact with various types of receptors (6–8). It has also been reported that Hsps such as Hsp70 and Hsc70 are expressed in the glycosphingolipid-enriched microdomain (GEM) on the cell surface (9,10).

Sialic acid-binding lectin (SBL) isolated from *Rana catesbeiana* oocytes was identified as a lectin, since SBL agglutinated certain types of tumor cells and the agglutination was inhibited by glycoprotein or ganglioside-containing sialic acid (11–13). Agglutination induced by SBL was observed only in tumor cells but not in normal red blood cells and fibroblasts (13). The amino acid sequence of SBL shows that it has homology to members of the RNase A superfamily, and it has been revealed that SBL has pyrimidine base-specific ribonuclease activity (14–17). The antitumor effect of SBL was reported using P388 and L1210 murine leukemia cells *in vitro* and sarcoma 180 cells and Ehrlich and Mep 2 ascites cells *in vivo*(18–20). We recently reported that SBL had a cytotoxic effect on various leukemia cells including MDR cells and showed that this cytotoxicity was induced through multiple apoptotic signaling pathways (21). Furthermore, data indicate that the SBL receptor (SBLR) may exist in the GEM on the cell surface (Tatsuta *et al*, unpublished data). In the present study, we investigated the involvement of Hsps in SBL-induced apoptosis, focusing on Hsp70 and Hsc70 that have been reported to exist in the GEM on the cell surface.

## Materials and methods

### Materials

SBL was isolated by sequential chromatography on Sephadex G-75, DEAE-cellulose, hydroxyapatite and SP-Sepharose as described previously (13). The anti-SBL antibody was established in our laboratory. The anti-caspase-3 antibody was purchased from BD Biosciences (Franklin Lakes, NJ, USA). The anti-Hsp70 and Hsc70 antibodies were purchased from Stressgen (Kampenhout, Belgium). The horseradish peroxidase (HRP)-conjugated anti-rabbit IgG antibody and the fluorescein isothiocyanate (FITC)-conjugated goat anti-rabbit antibody were purchased from Cedarlane (Hornby, Ontario, Canada). Quercetin was from Cayman Chemical Company (Ann Arbor, MI, USA).

### Cell culture

Mouse leukemia P388 cells were obtained from the Cell Resource Center of Biomedical Research, Institute of Development, Aging and Cancer, Tohoku University (Sendai, Japan). Cells were routinely maintained in RPMI-1640 medium (Nissui Pharmaceutical Co. Ltd., Tokyo, Japan) supplemented with 10% fetal calf serum (FCS), penicillin (100 U/ml) and streptomycin (100 μg/ml) at 37°C in a 95% air and 5% CO_2_ atmosphere.

### Detection of DNA fragmentation

The cells (2×10^5^ cells/ml) were cultured in 96-well plates (100 μl/well). After treatment with SBL, the cells were collected by centrifugation, washed with PBS, then lysed with cell lysis buffer [50 mM Tris-HCl (pH 6.8), 10 mM EDTA, 0.5% (w/v) sodium N-lauroylsarcosinate]. The samples were incubated with RNase A (final concentration, 500 μg/ml) for 30 min at 50°C, before being digested with proteinase K (final concentration, 500 μg/ml) for 30 min at 50°C. After the samples were electrophoresed on 1.8% agarose gel, DNA bands were visualized by ethidium bromide (EtBr) staining.

### Western blotting

Whole cell lysate was prepared with extraction buffer [10 mM Tris-HCl (pH 7.5), 150 mM NaCl, 1% Triton X-100, 5 mM EDTA (pH 8.0), 1 mM phenylmethylsulfonyl fluoride (PMSF) and 1 tablet/10 ml protease inhibitor cocktail (Roche Applied Science, Indianapolis, IN, USA)]. Lysates from the membrane, cytosol and nuclear fractions were prepared by ProteoExtract Subcellular Proteome Extraction kit (Merck Millipore, Billerica, MA, USA). Soluble proteins were collected, and concentrations were measured by the DC protein assay kit (Bio-Rad, Richmond, CA, USA) in accordance with the manufacturer’s instructions. Proteins were separated by SDS-PAGE and transferred to a polyvinylidene difluoride (PVDF) membrane (GE Healthcare, Little Chalfont, UK). The membrane was blocked using 5% fat-free skim milk for 1 h. After the membrane was washed with TBST [20 mM Tris-HCl (pH 7.6), 137 mM NaCl, 0.05% Tween-20], primary and secondary antibodies were added to the membrane, respectively. The proteins on the membrane were detected using ECL western blotting detection reagents (GE Healthcare). The intensity of the bands was calculated by Quantity One software (Bio-Rad).

### Flow cytometric analysis of SBLR, Hsp70 and Hsc70

For detection of SBLR, cells were treated with SBL for 30 min at 4°C, washed with PBS, and incubated with the anti-SBL antibody for 30 min at 4°C. Cells were washed with PBS, and then FITC-labeled anti-rabbit IgG was added and incubated for 30 min at 4°C. For detection of Hsp70 and Hsc70, cells were treated with anti-Hsp70 and anti-Hsc70 antibodies for 30 min at 4°C, washed with PBS and incubated with FITC-labeled anti-rabbit IgG for 30 min at 4°C, respectively. Fluorescence intensity was determined using a FACSCalibur flow cytometer (Becton-Dickinson).

### Reverse transcription and polymerase chain reaction (RT-PCR)

Total cellular RNA was isolated from the cells using TRIzol reagent (Invitrogen Life Technologies, Carlsbad, CA, USA). Reverse transcription (RT) was performed using ReverTra Ace (Toyobo, Osaka, Japan) with total RNA (1 μg) and oligo(dT)_12–18_ primers. The RT reaction mixture (1 μl) was subjected to PCR for 25, 40 and 30 cycles for GAPDH, Hsp70 and Hsc70, respectively, in a final volume of 20 μl of *Taq* DNA polymerase (1.25 U) (ABgene, Epsom, UK) and gene-specific forward and reverse primers for each gene. After initial denaturation at 94°C for 2 min, each of the cycles consisted of 94°C for 30 sec, 50°C for 30 sec and 72°C for 30 sec. The PCR products were separated on 1.5% agarose gel, and the bands were visualized with EtBr staining. The intensity of bands was calculated by Quantity One software.

### Measurement of cell viability

Cell viability was determined by the trypan blue dye exclusion assay. The cells (2×10^5^ cells/ml) were cultured with SBL (2 μM) and/or quercetin (5 μM) in 96-well plates. After treatment with SBL and/or quercetin, the cells were stained with 0.25% trypan blue, and both viable and nonviable cells were counted.

### Statistical analysis

Each experiment was performed at least in triplicate. The results are expressed as the means ± standard deviation. Statistical analysis was performed using unpaired Student’s t-tests; P<0.05 was considered to indicate a statistically significant difference.

## Results

### SBL-induced apoptosis in P388 cells

**Figure 1:**
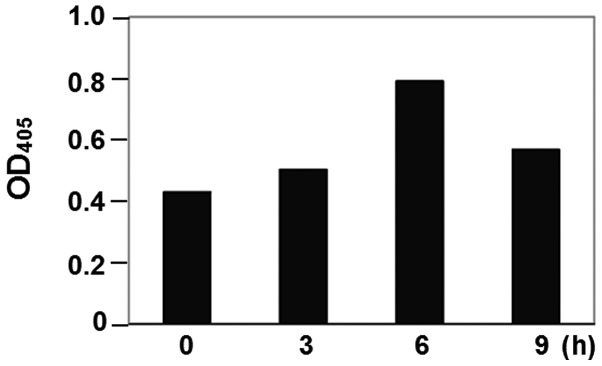
Effect of SBL on the activation of caspase-3 in P388 cells. Cells were treated with SBL (2 μM) for indicated time. Caspase-3 activity was examined by use of DEVD-pNA. SBL, sialic acid-binding lectin.

We recently reported that SBL induces apoptosis in various leukemia cell lines. In human leukemia Jurkat cells, typical apoptotic morphological change such as karyorrhexis, nuclear condensation and fragmentation, or apoptotic biological changes such as phosphatidylserine (PS) externalization, activation of caspases, DNA fragmentation were observed after treatment with SBL (21). In the present study, the apoptosis-inducing effect of SBL in P388 cells was analyzed by the detection of activated caspase-3. Caspase-3 activity was monitored by use of DEVD-pNA. The activity of caspase-3 was observed and maximized at 6 h of treatment (Fig. 1). As a concequence, SBL-induced apoptosis in P388 cells and the execution process may start as early as 6 h.

### Expression of SBLR, Hsp70 and Hsc70 on the P388 cell membrane

**Figure 2:**
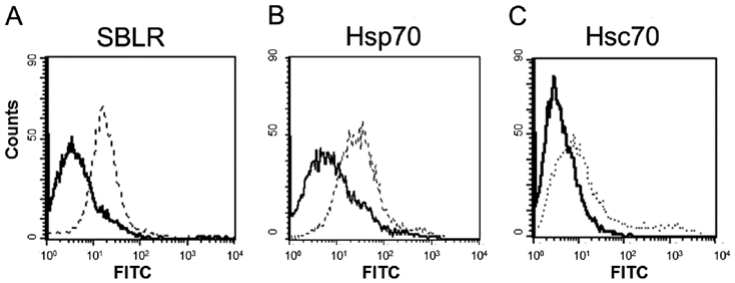
Flow cytometric analysis of heat shock proteins on the P388 cell surface. (A) SBLR, (B) Hsp70 and (C) Hsc70 on the P388 cell surface were analyzed by flow cytometry using respective antibodies (dotted line). Solid line indicates control cells for each experiment. SBLR, SBL receptor; SBL, sialic acid-binding lectin.

It has been suggested that SBL binds to the cell membrane to exert its antiproliferative effects which indicates the existence of SBLR. We analyzed the involvement of Hsps in SBL-induced apoptosis. We analyzed the expression of SBLR, Hsp70 and Hsc70 on the cell membrane by flow cytometric analysis. The results showed that both Hsp70 and Hsc70 were expressed on the cell membrane as well as SBLR (Fig. 2).

### Distribution of Hsp70 and Hsc70 in SBL-treated P388 cells

**Figure 3:**
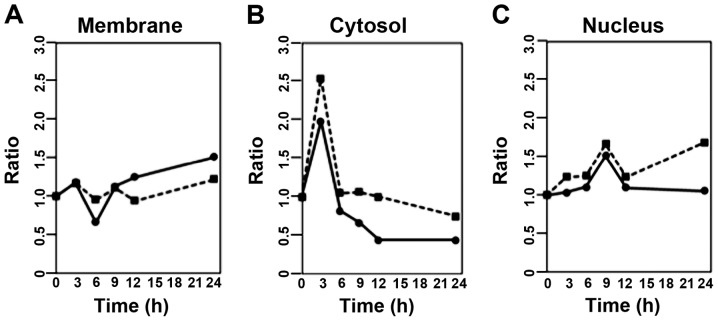
Distribution of Hsp70 and Hsc70 in the SBL-treated P388 cells. Each fraction [(A) membrane/organelle; (B) cytosol and (C) nuclear] was extracted from SBL-treated P388 cells. The levels of Hsps were detected by western blot analysis. Hsp70, solid line; Hsc70, dotted line. SBL, sialic acid-binding lectin; Hsps, heat shock proteins.

Our next experiment was designed to elucidate the localization of Hsp70 and Hsc70 after stimulation of SBL in P388 cells. After treatment with SBL, the cells were lysed and fractionated into the membrane, cytosol and nuclear fractions, and then the amounts of Hsp70 or Hsc70 were analyzed by western blotting. The results showed that Hsp70 in the membrane fraction at 6 h of treatment was decreased 34% when compared to the 0 h-control and then gradually increased (Fig. 3A). In the cytosol fraction, both Hsp70 and Hsc70 were transiently increased at 3 h of treatment, and a transient increase was also observed in the nuclear fraction at 9 h of treatment (Fig. 3B and C). After treatment with SBL, the localization of Hsp70 and Hsc70 was altered when compared to that in the cells without SBL treatment. Notably, SBL-induced activation of caspase-3 began to be observed, just after the amounts of Hsp70 and Hsc70 in the cytosol fraction reached maximum levels.

### Effect of quercetin on expression of Hsp70 or Hsc70

**Figure 4:**
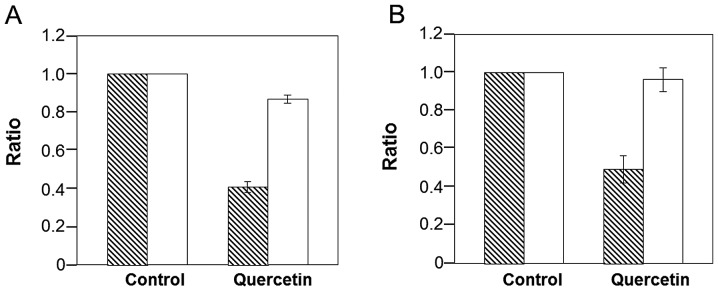
Effect of quercetin on mRNA expression and protein levels of Hsp70 and Hsc70. Cells were treated with quercetin (5 μM) for 12 h. Expression of Hsp70 (striped column) and Hsc70 (white column) at the (A) mRNA and (B) protein level was determined by RT-PCR and western blot analysis, respectively.

To clarify the relationship between Hsp70 and Hsc70 in SBL-induced apoptosis, we treated P388 cells with quercetin, a known bioflavonoid which decreases the expression of Hsp70. Treatment with quercetin for 12 h resulted in a 60% reduction in Hsp70 at the mRNA level and a 50% reduction at the protein level (Fig. 4). By contrast, quercetin did not affect the expression of Hsc70 at the mRNA or protein level.

### Effect of quercetin on the binding of SBL to P388 cells

As we confirmed that quercetin decreases the expression of Hsp70, sialidase treatment of cells was found to abolish tumor cell agglutination and the antiproliferative effect induced by SBL, and the existence of SBLR in the GEM on the cell surface has been suggested (13). In the present study, we investigated the possible involvement of Hsp70 and Hsc70 known Hsps which exist on the GEM, in SBL-induced apoptosis.

**Figure 5:**
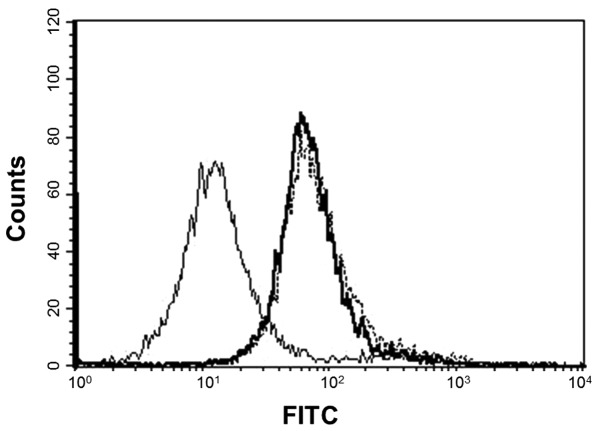
Effect of quercetin on the binding of SBL to P388 cells. Cells were treated with (bold line) or without (dotted line) quercetin (5 μM) for 12 h. After treatment with SBL and the anti-SBL antibody as described in Materials and methods, the cells were analyzed by FACSCalibur. Control, thin black line. SBL, sialic acid-binding lectin.

**Figure 6:**
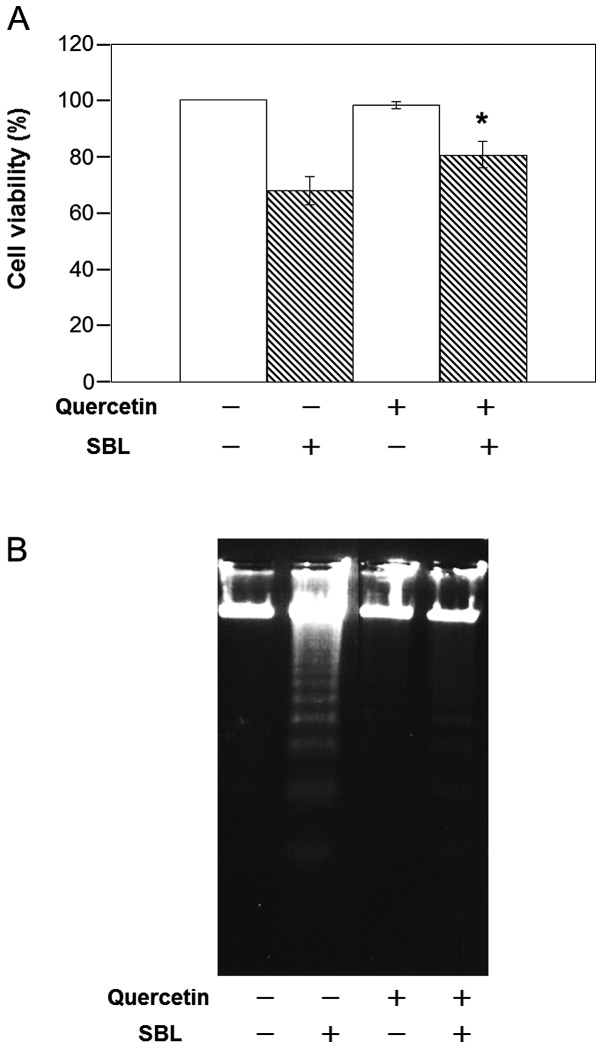
Effect of quercetin on SBL-induced cytotoxicity. After pretreatment with quercetin (5 μM) for 12 h, the cells were incubated with SBL (2 μM) for 24 h. (A) The viable cells were counted by trypan blue dye exclusion assay ^*^P<0.05 vs. SBL alone. (B) Agarose gel electrophoresis of DNA extracted from SBL-treated P388 cells. SBL, sialic acid-binding lectin.

We found that Hsp70 and Hsc70 were expressed on the P388 cell surface as well as SBLR (Fig. 2). Distribution of Hsp70 and Hsc70 was analyzed after treatment with SBL, and expression of Hsp70 and Hsc70 in the cytosol was dramatically increased immediately prior to the execution of apoptosis in SBL-treated P388 cells (Figs. 1 and 3). Next we studied the functional relationship of Hsps and the cytotoxic activity of SBL by the use of quercetin. Quercetin was previously found to decrease the expression of Hsp70 at the mRNA level by inhibiting the activation of HSF1 (23). The expression of Hsp70 was decreased at both the mRNA and protein levels in the quercetin-treated cells (Fig. 4). We found that binding of SBL to P388 cells was not affected by a decrease in Hsp70 expression by quercetin. The result indicates that Hsp70 itself may not be the receptor for SBL (Fig. 5). However, further study revealed that a decrease in Hsp70 by quercetin inhibited both SBL-induced cytotoxicity and apoptosis (Fig. 6), suggesting the important role that Hsp70 plays in SBL-induced apoptosis.

Various reports have indicated a relationship between Hsps, particularly the Hsp70 family, and various receptors on the cell surface. It was reported that Hsp70 and Hsc70 possibly interact with CD14, CD40 and the toll-like receptor family members (24–26). Notably, Guerrero and Moreno (27) reported that Hsc70 and integrin αvβ3 formed a complex in the GEM and act as a receptor for rotavirus and may participate in the process of adsorption and penetration of the viruses into cells. In the present study, we showed that binding of SBL was not affected by a decrease in the expression of Hsp70, but an attenuated induction of apoptosis was noted. It is possible that Hsps on the P388 cell surface may interact with SBLR or participate in the penetration of SBL into cells, and may affect the cytotoxicity of SBL, as cell susceptibility to RNase can be affected by binding as well as the internalization or translocation of RNases as described above. Since the expression of Hsc70 was not affected by quercetin, studies to clarify the possible involvement of Hsc70 or other Hsps in the function of SBL will be undertaken.

In summary, the present study demonstrated that Hsp70 and Hsc70 are expressed on the P388 cell surface as well as SBLR, and their expression levels are markedly increased in the cytosol immediately prior to the execution of apoptosis following SBL treatment. A functional study of Hsp70 revealed that decreased expression of Hsp70 suppressed the apoptosis induced by SBL. It is suggested, for the first time, that Hsps participate in the antitumor effect of cytotoxic RNases.
